# Medical Department University of Texas

**Published:** 1892-05

**Authors:** 


### MEDICAL DEPARTMENT UNIVERSITY OF TEXAS.

### COMMENCEMENT EXERCISES.

The commencement exercises of the Texas Medical College at the close of its first session were held in Galveston, Friday evening, April 21st. Of twenty-four matriculates there were three who had completed the course and received the degree of M. D. These gentlemen should feel very proud of being the first graduates of this high grade State school, a school destined to take its place high up in the list of educational institutions of America.

The degree of Doctor of Medicine was conferred by Dr. T. D. Wooten, the venerable and beloved President of the Board of University Regents, who has done so much for the cause of education in Texas. His remarks were brief, but most impressive, and doubtless sank deep into the hearts of the three young disciples. They evidently go forth duly impressed with the gravity of the responsibility this diploma lays upon their shoulders. Indeed, Professor Clopton's remarks in the closing address were almost enough to frighten a young man into turning back and retreating before the dangers and hardship depicted as awaiting him who *does his duty* as a practitioner.

Dr. Clopton said, in closing:

Have you chosen the profession of medicine alone because it may become a channel for the accumulation of money? If so, abandon it at the threshold. You are nothing but a tradesman, and there are vocations far better and more congenial to the money changer. Your alma mater expects nothing of any student upon whom she may confer the degree of Doctor of Medicine who measures his professional success by the single standard of money. Do you think the life an easy one, in which you may win honors without work? The life of the physician is one of hardships, self-denial and incessant labor, mental and physical. If you are not made of the stuff which makes doctors, your professional career will be short. You will desert early in the fight. You want the cardinal requisites, energy, devotion, character. To you who have chosen our profession from higher motives, that rise above self, and money, and ease, we extend the right hand of fellowship. We know already that your professional life will reflect honor upon your alma mater. As one whose best life has been spent in professional work, I warn you your life will be full of trials, self-denial and disappointment. At times, perhaps, you will almost despair, but there is an inspiration in the life which will uphold you, and beckon onward—the inspiration which moves the philanthropist to give his time and money to relieve suffering humanity; the inspiration which upholds the minister of the Christian gospel to endure unto the end for His cause sake. The same sentiment of devotion to the profession must inspire you that moved David, the psalmist, to exclaim, "I had rather be a doorkeeper in the house of my God than a dweller in the tents of Israel." Take the motto of the Douglas as your own: "Ready, O ready!" Ready to obey the call of duty, whether it summons you to the palace or the hut; as ready to peril your life to stay the pestilence as to treat the non-infectious malady in which there is no personal danger. Neither wife nor children must deter you from your duty. Your profession is jealous of a rival. Are you ready for all this, should it come? If not, you are not well prepared for your professional life, no matter how high your intellectual qualifications. You may succeed in a worldly sense, but in your soul you will be a groveling coward. Excuse me if in closing I indulge in the sentiments which compel me to speak of myself. What I shall say may sound egotistical, but I trust my audience will believe it is said in humility. With the experience of a number of years which my life work has given me, if I could stand at the threshold of my responsible life, with a prophet's vision as to its ending, and was given the choice of various pursuits, merchant, politician or physician, and I knew in the first I would in the end realize great wealth, and in the second high official position, with the plaudits of the people and in the last a knowledge of work skillfully done, of responsibilities boldly assumed, of duties faithfully discharged, of human life saved from impending death, I would choose the profession of which I have so long been an humble but devoted member. What is gold? What is political preferment? All vanity and vexation of spirit, says the wise man; but the discharge of duty and responsibility in the high calling of the physician, of restoring to health and usefulness a human being on whom death was impending, is not vanity or vexation of spirit, it is the priceless cord that binds our mortality to the highest immortality.

"When our souls shall leave this dwelling,
The glory of one fair and virtuous act
Is above all the scutcheons on our shields
Or silken banners o'er us."

The graduates were Thomas Flavin, who delivered the valedictory, H. T. Guinn, and I. T. Hendrick. We regret we did dot get the address of either, nor learn what community will be favored with their beginning skill.

Prof. J. F. Y. Paine, Dean of the Faculty, delivered an address which for ability, statistical value and comparison was remarkable, and should be read not only by every physician in Texas, but by the people, especially by the law makers. It is to be regretted that it cannot be reproduced here, on account of its length. The following extracts are taken from the *Galveston News*.

The dependence of the common welfare on medical education, and the interest of the public in the qualifications of men to whom their lives may be committed, prompt me to present a few facts relating to this important subject:

There are at the present time in the United States 135 medical colleges of all kinds. This number has not materially changed during the last ten years. New schools have been established, but a corresponding number have gone out of being. The geographical distribution of these institutions is by no means uniform, twelve being located in New York, fourteen in Missouri and fifteen in Ohio, while Alabama, Connecticut, Louisiana, New Hampshire, North Carolina, South Carolina, Texas and Vermont have but one each. Very little progress was made in medical education during the half century from 1830 to 1880. Two annual courses of lectures, the second a repetition of the first, and a short period of reading under the tutorage of a preceptor, constituted the sum of didactic instruction. First and second course students sat side by side, listened to the annual iteration of facts, saw from year to year the same anatomical and physiological models and preparations and viewed demonstrations on the same old manakins, which were considered sufficient to make a skillful physician of an illiterate booby as well as of a man with academic training. Twenty years ago there was not a medical school in the United States, of which I have knowledge, that required attendance upon more than two courses of lectures before graduation, or exacted a proper preliminary education as a condition to matriculation. The spirit of progress, however, which is characteristic of the nineteenth century, has lent inspiration to medicine, and that science is to-day in line of march, touching elbows with other departments of knowledge.

A review of the changes for the better since 1882 furnishes ample cause for congratulation. In that year the colleges requiring educational qualifications for matriculation numbered 45, against 129 that did so in 1891. In 1882 only 22 colleges required attendance upon three courses of lectures before graduation; to-day 85 impose that restriction. The duration of the lecture terms has been gradually increased from an average of five and a half months to six and a half months (in eight schools the time was only four months), 111 colleges now have sessions of six months or more, when in 1882 there were but 42 which had such long terms. The better class of schools now embrace hygiene and jurisprudence in their curriculum, and students are required to do practical work in the laboratories of chemistry, physiology, histology, pathology, bacteriology and pharmacology. But the movement of higher medical education has not stopped with this. Four colleges will soon exact four courses of six to nine months each.

The rules and curriculum have been arranged conformably to the demands of higher medical education and are as follows:

Candidates for admission (matriculation) are required, first, to write an essay of about 300 words in length as a test of orthography and grammar; second, to pass an examination in elementary physics. A candidate who has received a collegiate degree or passed the matriculation examination of a recognized college, or has a certificate covering the required subjects from a recognized normal or high school, may enter without examination. Students who have attended one course in a medical school (not homeopathic or electic), are admitted to the second year of the university course upon passing a satisfactory examination in osteology, descriptive anatomy, general chemistry, materia medica and pharmacy and the elements of general pathology. Students who have attended two courses in a regular medical school are admitted to the third year upon passing a satisfactory examination in topographical anatomy, general and medical chemistry, materia medica and therapeutics, physiology and the elements of general pathology. Graduates of regular medical schools in good standing are admitted to the third year without examination. Graduates of colleges of pharmacy in good standing are admitted to the second year upon passing the matriculation examination only.

The course of study extends over three years, which will be graded as follows:

First year—Didactic lectures upon: 1, osteology and descriptive anatomy; 2, physiology; 3, physics and general chemistry; 4, materia medica; 5, general pathology. Practical work in: 1, anatomy; 2, histology; 3, chemistry.

Second year—Didactic lectures upon: 1, topographical anatomy; 2, physiology; 3, chemistry; 4, materia medica and therapeutics; 5, pathology and practical medicine; 6, principles and practice of surgery; 7, obstetrics. Practical work in: 1, anatomy; 2, pathological histology; 3, medical chemistry. Chemical lectures in the John Sealy hospital in: 1, general surgery; 2, general medicine: 3, cases in obstetrics.

Third year—Didactic lectures in: 1, materia medica and therapeutics; 2, practical medicine; 3, general surgery; 4, obstetrics and gynecology; 5, special pathological anatomy. Practical work in: 1, clinical microscopy; 2, bacteriology and morbid anatomy; 3, chemistry of ptomaines and other bacterial products: Clinics in: 1, general surgery; 2, general medicine; 3, nervous diseases; 4, diseases of skin, venereal and genito-urinary diseases; 5, obstetrics and gynecology; 6, diseases of the eye, ear and throat; 7, diseases of children; 8, diseases of the bones and joints; 9, lectures upon medical jurisprudence.

Methods of instruction are by didactic lectures, practical demonstrations, bedside clinics and surgical operations. Recitations keep pace with the lectures, and students are marked according to their proficiency. Term marks are reviewed at the close of the session, and the average made affects the result of the final examination. The manner of grading is similar to that used by all the branches of the University. The grading of the courses of study is rigidly enforced, and students are required to devote themselves exclusively to the classes to which they belong. Under this restriction first-year men are limited to the study of fundamental branches of medical science, much of their time being spent in practical work in the laboratories, where they acquire familiarity by touch as well as by sight with every part of the human mechanism and with every agent that is brought in relation with it.

This system does not encourage our students to engage in practice after one year's study, a custom which, though much in vogue, can not be too emphatically condemned as an imposition upon society, and a travesty upon the medical profession. The most ample facilities, however, are afforded second and third course students for clinical study, daily clinics at the hospital being given throughout the entire session by members of the faculty. The number of patients available for clinical study is not so large as in some of the hospitals in more populous cities, yet there is always at hand a rich variety of cases, representing almost all forms of medical ailments peculiar to this climate, and a wide range of surgical afflictions and accidents.

The catalogues of twenty medical colleges for 1890 contain the names of 493 Texas students, who attended lectures in those institutions in that year. Of that number eighty-eight were registered in the six schools of higher grade, and 405 were distribnted among the others, where the tuition was cheaper, the terms of study shorter, and the graduation easier.

This exposition of facts leads to the conclusion that there has been a decided advance in the medical education during the last decade as regards both the methods of instruction and requirements for the degree in medicine; that the general ratio of students to population has not materially increased; that the general proportion of graduates to students has diminished; that the schools having the highest per cent. of graduates to students furnish the largest number of rejected applicants before the State examining boards; that the cost of tuition varies with the standing of the schools, and the advantages they afford, and that the large number of students leaving the State annually seems to indicate the necessity of a properly organized and thoroughly equipped medical school at home.

The public need to be educated to a just appreciation of the medical qualification to make a proper discrimination between true science and the assumption of ignorance and empiricism, and to offer every encouragement and facility for the acquisition of thorough medical education. "The end of medicine," says Aristotle, "is health." Consider what that means; not merely healing the sick, but teaching the people how not to be sick.

The faculty stand committed to devote their time, energies, and what of talent and ability they possess to the advancement of medical education, and to the making of this institution what its projectors intended it should be—a source of pride to the State, a credit to the profession of medicine, and a blessing to mankind.

The profession of Texas are to be sincerely congratulated both upon the successful inauguration of this college under such flattering auspices and upon the very high stand taken in the beginning. And no less are the thanks of the entire State due not only to the Board of Regents who have brought about so grand a consummation of this long cherished scheme, but to the gentlemen of the Faculty who have, one and all, so honorably acquitted themselves.

